# Valorization of Stale Bread and Sunflower Spent Oil via Solid State Fermentation Using Food-Grade Filamentous Fungi

**DOI:** 10.3390/biotech15030048

**Published:** 2026-06-28

**Authors:** Vahid Abbasi, Francisca P. Martínez-Antequera, Hadel Al-Roubai, Rahmo Abukar, Amir Mahboubi Soufiani

**Affiliations:** 1 Swedish Centre for Resource Recovery, University of Borås, 501 90 Borås, Sweden; vahid.abbasi@hb.se (V.A.); alrubaihadel@gmail.com (H.A.-R.); abukarrahmo1@gmail.com (R.A.); amir.mahboubi_soufiani@hb.se (A.M.S.); 2 Research Center on Intensive Mediterranean Agroecosystems and Agri-Food Biotechnology (CIAIMBITAL), Department of Biology and Geology, Faculty of Experimental Sciences, University of Almería, 04120 Almería, Spain

**Keywords:** filamentous fungi, solid state fermentation, circular bioeconomy, waste valorization, mycoprotein

## Abstract

Global food waste management necessitates circular bioeconomy solutions to transform organic residues into high-value nutrients to address nutritional demands. This study investigated the valorization of two abundant waste streams, stale bread and sunflower oil through solid state fermentation using food-grade filamentous fungi. Three strains, *Neurospora intermedia*, *Aspergillus oryzae* and *Rhizopus oryzae* were evaluated for the bioconversion of stale bread. Oil supplementation levels of 10, 20 and 30% (g/100 g dry matter) using both fresh and spent sunflower oil were tested to assess changes in proximate composition, characterizing fungal growth dynamics and mycelial development. Furthermore, modifications in fatty acid profiles and hydrolytic enzyme activities were analyzed to determine species responses to oil source and concentration. The results demonstrated that *N. intermedia* achieved peak protein levels of 36% (g/100 g) alongside efficient starch catabolism, while 10% fresh oil supplementation induced a significant protein increase (26%) in *A. oryzae*. Regarding lipid accumulation, 10% spent oil supported higher fat content in *R. oryzae* (19%) compared to fresh oil (17%). PUFA/SFA ratio reached its maximum in *A. oryzae* with the highest of 5.91 ± 0.56 under 10% fresh oil. Enzymatic analysis identified *A. oryzae* as the most efficient lipase producer, reaching a maximum activity of approximately 0.10 U/g at 10% spent oil supplementation. Conversely, *R. oryzae* lipase activity peaked at 20% supplementation (0.08 U/g), reflecting its high capacity for lipid accumulation. These findings establish a potent bioprocess for upcycling mixed food wastes into enhanced functional ingredients for sustainable food and feed systems.

## 1. Introduction

Global food waste is responsible for an estimated 8–10% of total greenhouse gas emissions [1]. This situation demonstrates the urgent need to make a transition toward a circular bioeconomy by efficiently valorizing organic waste streams into value-added bioproducts. Stale bread represents a major component of wasted food, with annual generation in Sweden alone exceeding 80,000 tons [2]. In parallel, approximately 24 million metric tons of spent oil are generated annually by food production industries worldwide, presenting environmental and management challenges [3]. Significantly, a considerable fraction of these materials is lost prior to consumer consumption, specifically during the processing and distribution stages of the supply chain. The transformation of waste streams into high value product through biological platforms including microalgae, bacteria and filamentous fungi offers a strategic opportunity to simultaneously mitigate global waste management issues and address the growing scarcity of essential nutrients [4,5].

Filamentous fungi are increasingly recognized as robust whole-cell biocatalysts that employ diverse enzymatic mechanisms to valorize various organic waste streams into protein-rich biomass, frequently categorized as single-cell protein (SCP) [6,7]. This bioconversion is a key step in making sustainable, food-grade fungal products, which are often called mycoprotein with a desirable balance of essential amino acids and dietary fibers for food and feed applications [8,9]. For instance, species belonging to the genera Neurospora and Rhizopus are globally valued for their efficacy in converting starch-rich agricultural side streams into protein-rich biomass, with protein levels reaching approximately 57.6% and 50.9% on a dry weight, respectively [10]. This metabolic capability has been traditionally exploited in the production of various Indonesian fermented foods like Oncom [11].

In addition to protein synthesis, filamentous fungi are capable of accumulating substantial lipids ranging from 20 to 80% of their dry biomass, which qualify them as single-cell oil (SCO) [12]. Unlike many traditional vegetable oils, fungal lipids are typically enriched with polyunsaturated fatty acids (PUFAs), which are highly valued for human health [13,14]. Genera such as Mucor, Rhizopus, and Aspergillus exhibited specialized enzymatic machinery including desaturases and elongases, which allow them to bioconvert saturated or monounsaturated fatty acids into nutritionally essential long-chain PUFAs, such as arachidonic acid (ARA) and eicosapentaenoic acid (EPA) [15,16]. Functioning as cellular biorefineries, these microorganisms synthesize intracellular lipids through de novo pathways from carbohydrate sources as well as the ex novo accumulation and metabolic modification of external lipid sources [12,17]. This metabolic adaptability facilitates the enrichment of biomass with high-value microbial oils, providing a sustainable alternative to conventional lipid sources.

The implementation of an optimal fermentation strategy is crucial for efficiency and sustainability of the bioconversion process. Solid-state fermentation (SSF) has emerged as a particularly effective approach for the bioconversion of solid residues, as it utilizes low-moisture substrates, reduces energy consumption, and minimizes wastewater generation [18,19]. Stale bread is considered an ideal solid substrate for SSF due to its high starch content, which serves as a readily accessible carbon source for filamentous fungi. Previous studies have successfully utilized substrates like wheat bran or coconut oil cake supplemented with lipid sources such as linseed oil (at concentrations around 1% *v*/*v*) to stimulate fungal growth and facilitate the enrichment of the final biomass with polyunsaturated fatty acids [20,21]. These stable fermented bioproducts are multifunctional, with applications as protein-rich ingredient in feed and food formulations.

Although filamentous fungi have been widely recognized for their potential in PUFA production and food-waste bioconversion, previous research has often relied on submerged fermentation and focused on single waste streams [22,23]. The performance of different fungal species in the solid-state fermentation of complex substrates such as stale bread supplemented with lipid-rich residues has not been explored. The interaction of oil type and oil concentration in determining biomass composition, fatty acid transformation, and hydrolytic enzyme secretion in fungal strains remains insufficiently understood [24,25]. Consequently, further investigation is needed to develop efficient SSF-based processes capable of simultaneously valorizing bread and spent oil into nutritionally enhanced fungal biomass.

Therefore, this study aimed to evaluate the solid-state fermentation of stale bread using three filamentous fungi including *Neurospora intermedia*, *Aspergillus oryzae*, and *Rhizopus oryzae* supplemented with both fresh and spent sunflower oil. The study examined how fungal species and oil supplementation influence biomass composition, including crude protein, crude fat, crude fiber, and fatty acid profiles. In addition, key hydrolytic enzymes, including protease, lipase, and chitinase activities, were quantified to characterize the metabolic capacities of each fungal strain in protein synthesis and fat degradation under SSF conditions.

## 2. Materials and Methods

### 2.1. Substrate Preparation

Stale bread, obtained from a local store in Sweden (ICA, Knalleland, Borås, Sweden), served as the substrate for solid-state fermentation. The bread was cut into pieces and air-dried to achieve a moisture content of 11.7 ± 2.3%. It was then milled using a laboratory grinder fitted with a 2 mm mesh size. The milled bread was further dried in an oven at 60 °C for 12 h. Moisture content was subsequently determined using a moisture analyzer (DBS 60-3, Kern & Sohn GmbH, Balingen, Germany), resulting in a final value of 1.2 ± 0.1% on a dry weight basis. The prepared substrate was sealed and stored at 4 °C until required for fermentation. Fresh and spent sunflower oil was obtained from a local restaurant (Restaurang Balder, Borås, Sweden). Both oils were sterilized by autoclaving prior to their use in the fermentation process.

### 2.2. Fungal Strains

The food-grade filamentous fungi, *Rhizopus oryzae* CCUG 28,958 (Culture collection University of Gothenburg, Sweden), *Aspergillus oryzae* CBS 819.72 and *Neurospora intermedia* CBS 131.92 (Centraalbureau voor Schimmelcultures, Westerdijk Fungal Biodiversity Institute, Utrecht, The Netherlands) were used. All fungal strains were grown on potato dextrose agar plates (PDA) that contain 4 g/L potato infusion, 20 g/L dextrose and 15 g/L agar. The *N. intermedia* strain was incubated for 3–4 days at 30 °C, whilst the *R. oryzae* and *A. oryzae* were incubated for 5–6 days at the same condition, following the described method [26]. The inoculum suspension was prepared by adding 20 mL of autoclaved distilled water to the PDA plates. A plastic spreader was then used to disperse and release the spores. The number of spores in the inoculum was counted using a Bürker chamber under a light microscope (Carl Zeiss Axiostar plus, Jena, Germany).

### 2.3. Solid State Fermentation (SSF)

Dried bread was crumbed to a particle size of approximately 2 mm, providing an intrinsic alveolar structure and adequate natural porosity to prevent bed compaction. This ensured proper aeration and hyphal penetration without need for inert support matrices. The substrate was autoclaved at 121 °C for 20 min. No additional nutrient supplements (e.g., external nitrogen sources) were added; stale bread served as the sole substrate in the control groups, while sunflower oil was the only supplement in the oil-treated groups. Inoculum spore suspension (3 mL, 3.2 × 10^5^ ± 0.5 spores/mL) was introduced into the substrate (10 g for each sample), and autoclaved water was then added to each treatment. The moisture content of all treatments was adjusted at the same level (54%). This value was selected based on preliminary optimization trials, ensuring the physical stability and proper consistency of the samples across all oil concentrations. Oil treatments were prepared with concentrations of 10, 20 and 30% (*w*/*v*) using fresh and spent oil supplementation. The inoculum, the substrate and the oil were thoroughly homogenized using a sterile plastic spreader. Control treatment without oil served as a baseline for comparison. All preparations were made under aseptic conditions. The inoculated plates were transferred to a climate chamber (HPP110eco, Memmert, Germany) set to maintain constant environmental parameters, including 50% relative humidity and 30 °C, for five days [27]. Following fermentation, the outcome (mycelium-bread) was dried in an oven dryer at 50 °C for 12 h and then milled into a fine meal using a laboratory milling device.

### 2.4. Analytical Methods

Crude protein content was determined using the Kjeldahl method (FOSS Kjeltec analyser) following the protocol [28]. Protein content was calculated by multiplying total nitrogen by a conversion factor of 5.89 according to Gmoser, Sintca [23]. Crude fat was quantified using an extraction unit (Soxtec ST 243, FOSS, Denmark) in accordance with AOAC Official Method 2003.05 [29]. Crude fiber was measured with a Fiber Analyzer ANKOM 200 according to AOCS Official Method Ba 6a-05. Sequential digestion carried with 0.255 N sulphuric acid and 0.255 N sodium hydroxide, followed by ashing to determine the organic fiber fraction by weight loss. Total ash content was determined by gravimetric dry ashing in accordance with AOAC Official Method 942.05. Freeze-dried samples were incinerated in a muffle furnace 550 °C for 4 h. The ash content was calculated based on the weight of the inorganic residue remaining after combustion. Total starch content was determined according to AOAC Method 996.11 via enzymatic hydrolysis. Samples were treated with thermostable α-amylase and amyloglucosidase to hydrolyse starch to D-glucose, which was then quantified using Waters Alliance 2695 HPLC system (Waters Corp., Milford, MA, USA). Detection was conducted at 490 nm. All samples were analyzed in triplicate.

The extraction of fatty acids was performed utilizing the Two-step Transesterification (2-TE) method [30]. Following the lipid extraction, methylation was performed utilizing sulfuric acid 2%. The methylated fatty acids were subsequently examined using gas chromatography (GC Clarus 550, Perkin-Elmer, Norwalk, CT, USA). A 1 µL sample was injected using a 15:1 split ratio at an inlet temperature of 250 °C. Nitrogen served as the carrier gas at a consistent flow rate of 1 mL/min. The oven temperature protocol consisted of an initial hold at 50 °C, followed by a ramp at 11.9 °C/min to 175 °C, a subsequent ramp at 1.9 °C/min to 230 °C, and a final hold for 5 min. Individual fatty acids identified by gas chromatography were classified as saturated fatty acids (SFA), monounsaturated fatty acids (MUFA), or polyunsaturated fatty acids (PUFA), and their relative proportions were expressed as percentages of total fatty acids.

### 2.5. Enzyme Activity Measurement

The enzymatic activities of all ingredients were analyzed in samples prepared by extracting 500 mg of each ingredient in 3 mL of distilled water. The mixture was kept at 4 °C for 2 h under continuous agitation using a rotary shaker, followed by centrifugation at 4000× *g* for 15 min to obtain the aqueous extract. For all enzymatic assays, the incubation of the extracts was carried out under the same conditions: 25 °C for 90 min. Acid protease activity was measured at pH 4.0 using hemoglobin as the substrate, following the method described by Anson [31]. Alkaline protease activity was determined at pH 8.0 using casein as the substrate, according to Walter [32]. Chitinase activity was evaluated by hydrolysis of 0.5% chitin from shrimp shells (Ref. C9752; Sigma-Aldrich, St. Louis, MO, USA) in Sodium Acetate Buffer 0.05 M at pH 5.0, resulting in the release of N-acetyl-D-glucosamine. The concentration of reducing sugars (N-acetyl-D-glucosamine) released during the reactions was quantified using 3,5-dinitrosalicylic acid (DNS), following the method of Miller [33], with standard curves constructed using known concentrations of N-acetyl-D-glucosamine. Lipase activity was determined using the β-naphthyl myristate colorimetric method adapted from Seligman and Nachlas [34]. Briefly, samples were incubated with β-naphthyl myristate in TRIS-HCl buffer (pH 8.0) supplemented with CaCl_2_ and sodium taurocholate. Following incubation, the released β-naphthol was coupled with Fast Blue BB salt to form an azo-dye. The reaction was terminated with trichloroacetic acid, and the resulting chromophore was stabilized in an ethyl acetate-ethanol solution. Absorbance was recorded at 540 nm using a spectrophotometer (GENESYS™, Thermo Fisher Scientific, Madison, WI, USA), with standard curves constructed using known concentrations of β-naphthol. All enzyme activity assays were performed in triplicate, and two blank controls with heat-inactivated enzyme extracts were included in each assay to correct for background absorbance. One unit of enzymatic activity (U) was defined as the amount of enzyme that releases 1 µmol of product per minute.

### 2.6. Statistical Analyses

Statistical analysis was conducted using a General Linear Model (GLM) to evaluate the main effects and the comprehensive interactions (two-way and three-way) between fungal strain, oil concentration, and oil type. Post hoc multiple comparisons were performed using Tukey’s honest significant difference (HSD) and Fisher’s least significant difference (LSD) tests, as appropriate, to identify statistical differences between treatment mean values. Fermentation and enzyme activity assays were performed in triplicate, while proximate analysis and fatty acid profiling were conducted in duplicate. All statistical procedures were executed using Minitab^®^ version 21.1.1 (©2022 Minitab, LLC, State College, PA, USA).

## 3. Results and Discussion

The mycelium-breads from solid state fermentations were analysed through proximate analysis to assess macronutrient dynamics and the efficiency of substrate upcycling. This was followed by a detailed characterization of lipid accumulation and fatty acid profile optimization, highlighting species-specific restructuring of exogenous oil. To provide a mechanistic understanding of these nutritional shifts, the secretion patterns of key hydrolytic enzymes were examined in relation to growth and nutrient mobilization.

### 3.1. Proximate Composition

The substrate’s proximate composition was significantly altered by the fermentation of stale bread, as detailed in Table 1. Statistical analysis confirmed that the bioconversion process was significantly influenced by the interaction of fungal strain, lipid concentration, and oil quality (*p* < 0.05).

Regarding the initial fermentation substrate, the theoretical carbon-to-nitrogen (C/N) ratio of the fermentation media was determined based on the elemental composition of the stale bread (62.0% starch and 2.1% total nitrogen) and the respective oil supplementation levels, according to standard conversion factors [35]. For the control treatment (0% oil), the medium presented 62.0% starch, 2.10% total nitrogen, and 33.86% total carbon, resulting in a C/N ratio of 16.12. With the addition of 10% oil, the values decreased proportionally to 55.8% starch, 1.89% nitrogen, and 38.10% carbon, shifting the C/N ratio to 20.16. At 20% oil supplementation, the medium contained 49.6% starch, 1.68% nitrogen, and 42.31% carbon, which increased the C/N ratio to 25.18. Finally, the highest supplementation level of 30% oil resulted in 43.4% starch, 1.47% nitrogen, and 46.51% carbon, yielding a maximum C/N ratio of 31.64.

#### 3.1.1. Crude Protein

The crude protein (CP) of the fermented biomass varied significantly among fungal species, oil type and supplementation levels (*p* < 0.05). All fermented treatments exhibited increase in CP compared to unfermented bread (11.5%). *N. intermedia* yielded highest CP (36%) among the oil-free controls followed by *R. oryzae* (26%) and *A. oryzae* (22%). Decreasing trend in CP was observed as oil supplementation increased from 10 to 30%. In *N. intermedia* protein content declined sharply from 36% in the oil-free control to 18% and 16% at 30% supplementation with fresh and spent oil, respectively. Similarly, *R. oryzae* showed progressively lower protein values with increasing oil concentration, reaching the lowest level of 12% under 30% fresh oil supplementation.

Filamentous fungi are well recognized for their ability to utilize diverse substrates and produce protein-rich biomass during fermentation [6]. However, the crude protein yield in solid-state fermentation (SSF) is highly dependent on the substrate and fungal species [36]. In the present study, the crude protein content obtained for the *N. intermedia* oil-free control was in agreement with earlier SSF studies employing bread as the sole substrate, which reported protein levels in the range of 35–36%. In contrast, the CP obtained for *A. oryzae* in the oil-free control represented a twofold increase compared to the 56% and 69% increases previously reported when using wheat bran and defatted rice bran, respectively [37].

A constant decreasing trend in CP was observed for most species as oil supplementation increased from 10% to 30%. For *N. intermedia*, protein content dropped from 36 to 18% and 16% at the 30% level for fresh and spent oil, respectively. The physical characteristics of the substrate have a direct influence on mycelium growth during SSF [38]. Hence, increasing the oil concentration induces physical modifications in heat transfer and oxygen accessibility which likely impacted mycelial development compared to the oil-free control group.

*R. oryzae* exhibited the lowest protein values at the maximum supplementation level, reaching 12% with 30% fresh oil. Beyond physical substrate modifications, this trend suggests a rapid metabolic shift wherein *R. oryzae* prioritizes lipid accumulation over protein synthesis in the presence of abundant exogenous fats. Such a redirection of metabolic flux aligns with reports by Hama, Tamalampudi [39], indicating that lipid supplementation can trigger a prioritization of lipid metabolism and storage at the expense of primary biosynthetic pathways.

A different trend was observed for *A. oryzae*, where the addition of 10% fresh oil induced a significant protein increase (26%) compared to its control (22%). This inductive effect is corroborated by evidence of the superior growth performance in lipid-enriched environments; for example, the biomass yield of *A. oryzae* has been shown to increase from 4 g/L to 16 g/L when supplemented with olive oil [40]. This capability is crucial for utilizing oils effectively at low concentrations act as metabolic stimulants for growth and metabolism [41].

Furthermore, fresh oil generally supported higher protein yield compared to spent oil across most treatments. For *N. intermedia*, supplementation with 10% fresh oil resulted 31% protein, compared to 27% with spent oil. The reduced protein yields associated with spent oil are attributable to the formation of secondary oxidation products, such as aldehydes (e.g., hexanal, benzaldehyde) and ketones (e.g., 2-undecanone), generated during thermal degradation [42]. These compounds have been demonstrated to inhibit fungal growth and spore germination, disrupt energy metabolism and ribosomal stability, and induce DNA damage, thereby impairing the overall metabolic capacity for protein synthesis and biomass accumulation [43].

#### 3.1.2. Crude Fiber

While mycelium growth inherently increased the crude fiber content of the substrate compared to the unfermented bread (1.5%), lipid supplementation significantly modulated this accumulation in a species-dependent manner. In *R. oryzae*, fiber levels were negatively impacted by oil addition, decreasing from 20% in the control to an experimental low of 7% at 30% oil supplementation. *N. intermedia* exhibited a similar downward trend, with fiber dropping from 24% to 11% under 30% spent oil treatment. Conversely, *A. oryzae* maintained markedly higher fiber concentrations (up to 32%) with 10% oil addition, indicating a more conservative structural modification strategy or a genus-specific cell wall composition.

The fungal cell wall is a complex structure primarily composed of fibrous polysaccharides, including glucans and chitin which support essential structural integrity and mechanical flexibility to the mycelia [44]. The dynamic nature of the cell wall facilitates adaptation and interaction with the solid-state environment [45]. In filamentous fungi, crude fiber measurements serve as an indirect index of mycelial growth, reflecting the accumulation of these structural polysaccharides [46]. It is important to note that the analytical method employed for the crude fiber analysis in this study involved sequential acid and base treatments (described in Section 2.4), a protocol standardly optimized for lignocellulosic materials. Consequently, the significantly lower fiber content observed in R. oryzae can be attributed to the specific structural composition of its cell wall compared to the Ascomycota strains.

The cell walls of Ascomycota, such as *N. intermedia* and *A. oryzae* are characterized by a high proportion of beta-glucans that link to chitin which increases overall cell wall rigidity [47]. These complex polysaccharide linkages likely enhance resistance to the acid-base digestion employed in crude fiber analysis. This structural variation likely explains the differences observed at statistically similar protein concentrations. For instance, both *A. oryzae* supplemented with 10% oil and the *R. oryzae* control yielded approximately 26% protein, while *R. oryzae* exhibited a notably lower crude fiber content (20%) compared to *A. oryzae* (32%).

#### 3.1.3. Starch

Fermentation resulted in a substantial depletion of the initial starch content (65% of dry matter) across all treatments. All fungal species showed a significant reduction in starch; however, the extent of depletion varied markedly among genera. At comparable crude protein concentrations of approximately 26% (specifically, *A. oryzae* with 10% fresh oil, *N. intermedia* with 10% spent oil, and *R. oryzae* without oil), *R. oryzae* retained significantly higher residual starch levels, ranging from 21% to 29%. In contrast, *N. intermedia* showed extensive starch depletion, with residual values consistently below 10% across all treatments.

These results indicate clear taxon-dependent differences in carbohydrate utilization efficiency during fermentation. Such differences are consistent with variations in the enzymatic capacity of filamentous fungi to hydrolyze complex carbohydrates. Filamentous fungi facilitate starch catabolism through the secretion of amylolytic enzymes, enabling the conversion of polysaccharides into simpler sugars for metabolic use.

In this context, it should be noted that amyloglucosidase or glucoamylase (EC 3.2.1.3) plays a key role in this process [48]. The amylolytic system typically operates in a two-step process: (1) liquefaction, where α-amylase (EC 3.2.1.1) randomly cleaves internal α-1,4 glycosidic bonds, reducing polymer viscosity and generating dextrins; and (2) saccharification, where glucoamylase acts as an exo-enzyme, releasing glucose units from non-reducing ends and hydrolyzing both α-1,4 and α-1,6 linkages [48]. This enzymatic framework provides a mechanistic explanation for the differential starch depletion observed among fungal strains.

From a metabolic perspective, the residual starch patterns, particularly in *R. oryzae*, suggest differences in substrate utilization efficiency among taxa. Starch has been reported as a highly suitable substrate for Mucoromycota. For example, in studies with *Cunninghamella echinulata* CCRC 31840, soluble starch was identified as the preferred carbon source for growth, resulting in higher biomass and increased total γ-linolenic acid (GLA) production compared to glucose as the sole carbon source [49,50]. Although the relative GLA content in lipids may be lower when using starch, absolute yields are enhanced due to greater biomass accumulation [49]. Similarly, other Mucorales such as *Mortierella* sp. also exhibit strong growth on starch-based media, although this preference is strain-dependent [48].

In addition, filamentous fungi can regulate enzyme secretion depending on environmental conditions such as pH, temperature, and nutrient availability, allowing efficient substrate utilization [51,52]. This regulatory flexibility enables partial preservation of carbohydrate reserves when alternative energy sources are available, particularly under lipid supplementation.

A similar but less pronounced pattern was observed in *A. oryzae* and *N. intermedia*. Both species showed their lowest residual starch in the oil-free control group (6%), indicating maximal carbohydrate consumption in the absence of lipids. However, supplementation with 10% oil significantly increased starch retention. In *A. oryzae*, starch nearly doubled to 12% with fresh oil and 11% with spent oil. Likewise, *N. intermedia* reached 9% residual starch under 10% spent oil supplementation. These results indicate a metabolic shift in which exogenous lipids act as an alternative energy source, thereby sparing carbohydrate reserves.

This lipid-induced metabolic shift is consistent with observations in other food-grade filamentous fungi, where vegetable oil supplementation enhances mycelial growth and biological efficiency (BE) [53]. For instance, in *Pleurotus ostreatus*, oil supplementation has been reported to achieve BE values as high as 109.43%, highlighting the role of lipids in improving substrate utilization efficiency.

From a process engineering perspective, the ability of filamentous fungi to simultaneously secrete amylolytic enzymes and convert released sugars into valuable metabolites (such as microbial lipids or mycoprotein) enables the development of consolidated bioprocesses (CBP) [48]. In CBP systems, starch hydrolysis and bioconversion occur in a single step, reducing or eliminating the need for external enzymatic treatment or thermal pre-processing.

Finally, carbon source prioritization in filamentous fungi is governed by regulatory mechanisms such as carbon catabolite repression (CCR), transcriptional regulation, and metabolic control pathways [54,55]. These mechanisms allow fungi to adapt to heterogeneous waste streams and optimize biomass production. In the present study, starch appears to be the preferred carbon source for *A. oryzae* and *N. intermedia*, as evidenced by crude protein and fiber as the mycelium growth markers. However, clear taxonomic differences remain: *R. oryzae* retained approximately 22% residual starch under control conditions (26% CP), whereas *N. intermedia* under comparable protein levels (10% fresh oil; 31% CP) showed much lower residual starch (9%), despite lipid availability.

#### 3.1.4. Crude Fat

In parallel with starch dynamics, crude fat content increased with oil supplementation across all treatments compared to stale bread (2.0%), regardless of fungal strain or oil type. In oil-free controls, clear strain-dependent differences were observed: *A. oryzae* and *N. intermedia* exhibited similar crude fat contents (3%), whereas *R. oryzae* accumulated significantly higher fat content (6%).

These results highlight that, in addition to carbohydrate metabolism, the fungal strains display distinct strategies for lipid assimilation and storage. The addition of fatty materials as co-substrates of sugars has been reported to enhance lipid accumulation in several microorganisms [56,57]. In some cases, microorganisms that do not produce appreciable lipid levels on sugar-based media significantly increase lipid accumulation when exogenous fats are supplied [57,58].

Oleaginous filamentous fungi have also demonstrated the ability to utilize starch-rich waste streams, such as potato processing residues, for lipid biosynthesis [59]. This metabolic efficiency is closely linked to the secretion of key amylolytic enzymes, including α-amylases and glucoamylases, which convert starch into simpler sugars that can subsequently be channeled into lipid biosynthesis pathways [51,60]. In this context, the present results indicate that *R. oryzae* exhibits a higher capacity for converting carbohydrates into lipids during solid-state fermentation of bread as a substrate.

Environmental parameters such as pH, temperature, and substrate moisture content also play a critical role in regulating enzyme secretion and lipid metabolism, thereby influencing overall process efficiency [61].

With 10% oil supplementation, the three fungal strains showed markedly different responses in crude fat accumulation. *A. oryzae* exhibited a relatively low lipid content (~6%), lower than the other strains, whereas *R. oryzae* showed the highest increase, reaching approximately 17%. This phenomenon is described as *ex novo* lipid accumulation, a term first introduced by [56]. Unlike de novo lipid synthesis from sugars, which is typically triggered by nitrogen limitation, *ex novo* lipid accumulation from hydrophobic substrates represents a primary anabolic activity that occurs concurrently with cell growth and is independent of nitrogen availability.

Although lipid accumulation is a key parameter, its interpretation alongside growth-related indicators such as crude protein and fiber provides a more comprehensive understanding of species-specific metabolic strategies. In *A. oryzae*, despite its lower lipid content, protein (26.28%) and fiber (32%) increased substantially compared to the control group. These results are consistent with reports indicating that *A. oryzae* can actively catabolize lipids to support mycelial development [62,63].

In contrast, despite its higher lipid accumulation, *R. oryzae* showed a significant decrease in protein content following oil supplementation. Similar trends were observed for crude fiber, suggesting that growth of *R. oryzae* was limited by oil addition even at low concentrations. Oxygen availability is critical for *R. oryzae* growth, and oxygen limitation can lead to a metabolic shift towards ethanol production and eventual loss of viability [64]. As oil concentration increased up to 30%, a clear transition from active growth to a storage- or inhibition-dominated metabolic state was observed. Under these conditions, lipid accumulation reached maximum values of 39% for *N. intermedia* and 37% for *R. oryzae.* These high accumulation levels on sunflower oil are consistent with reports for Mucorales, where lipid contents in biomass can reach 42.7–65.8% [65].

The nature of the oil source also influenced these accumulation patterns. At the 10% supplementation level, spent oil supported higher lipid retention in *R. oryzae* (19%) compared to fresh oil (17%). This is likely due to the higher initial concentration of free fatty acids (FFAs) in spent oil, which facilitates faster passive diffusion into the mycelium compared to fresh triacylglycerols [56,65]. The increased lipid availability likely elevated the carbon-to-nitrogen ratio, promoting a metabolic shift from biomass formation towards lipid storage, while residual oil further diluted the relative proportions of protein and fiber.

#### 3.1.5. Fatty Acid Characterization

The fatty acid profile of the fermented biomass showed clear strain dependence and marked differences compared with the initial bread substrate (Table 2). The unfermented bread exhibited a profile composed of 12% SFA, 42% MUFA, and 45% PUFA, which is consistent with typical wheat-based formulations where polyunsaturated fatty acids—mainly linoleic acid (C18:2n-6)—represent the dominant fraction [66].

These baseline values provide a reference for evaluating how fungal metabolism and oil supplementation reshape lipid composition during solid-state fermentation. In oil-free conditions, *A. oryzae* and *R. oryzae* showed a substantial enrichment in PUFA content (53.31% and 55.29%, respectively), accompanied by a reduction in MUFA compared with the original bread. This shift reflects strong endogenous desaturase activity and the conversion of oleic acid into more unsaturated derivatives such as linoleic acid [67,68]. This process can be interpreted as a form of “fat biomodification”, in which the fungal enzymatic machinery actively restructures the available lipid pool to meet physiological and membrane requirements [56].

In the case of *R. oryzae*, and more broadly Mucorales, it is important to highlight their well-established capacity to produce γ-linolenic acid (GLA, C18:3n-6) [49,50]. Although both *A. oryzae* and *R. oryzae* display high desaturase activity, the allocation of fatty acids differs among species and is strongly influenced by substrate composition. In general, unsaturated fatty acids are preferentially directed toward membrane synthesis and growth-associated metabolism. In this regard, fatty acid profiling (Table 2) indicates that although PUFA levels in *N. intermedia* were lower than in the other strains, its overall biomass productivity was higher, suggesting a distinct metabolic prioritization.

Building on this baseline, sunflower oil supplementation markedly altered the fatty acid distribution across all strains. With oil addition, MUFA became the dominant fraction in all experimental conditions. At 10% oil supplementation, SFA content decreased in all strains, while MUFA levels increased correspondingly. Regarding PUFA, *N. intermedia* was the only strain that maintained relatively stable levels after 10% oil addition, whereas both *A. oryzae* and *R. oryzae* showed a clear decrease.

The stability of PUFA content in *N. intermedia* can be attributed to the similarity between its intrinsic lipid profile (approximately 29%) and the exogenous sunflower oil composition. In addition, this behavior suggests a coordinated regulation of lipid storage and utilization, potentially optimizing energy efficiency, in a manner comparable to mechanisms described in *Neurospora crassa* [67].

As oil concentration increased up to 30%, MUFA reached maximum values with *R. oryzae* achieving 62% under spent oil conditions and *N. intermedia* reaching 61% under fresh oil supplementation. These maxima reflect the progressive incorporation and dominance of monounsaturated fatty acids derived directly from the sunflower oil.

From a functional perspective, saturated fatty acids contribute to membrane rigidity and energy storage, whereas polyunsaturated fatty acids are essential for maintaining membrane fluidity and supporting fungal growth [69]. Accordingly, the PUFA/SFA ratio represents a key indicator of fungal lipid remodeling mediated by desaturase activity. This ratio reached its highest value in A. oryzae (5.9) under 10% fresh oil supplementation. Tukey’s HSD analysis confirmed this as a statistically distinct group (“a”), indicating a superior capacity for fat biomodification [56], characterised by efficient restructuring of exogenous lipids into more unsaturated forms.

In contrast, *R. oryzae* exhibited a pronounced shift under oil supplementation, with PUFA proportions decreasing from its intrinsic baseline (~55%) to 29–36%. This trend suggests a transition towards a storage-dominated metabolic state, in which the final lipid profile becomes increasingly determined by the composition of the exogenous oil rather than endogenous biosynthetic activity.

Furthermore, spent oil supplementation consistently promoted higher SFA accumulation, peaking at 12% in *N. intermedia* (10%), while yielding lower PUFA levels than fresh oil. This trend is likely attributable to the thermal oxidative degradation of linoleic acid during previous cooking cycles, which reduces the availability of unsaturated substrates for fungal bioconversion and increases the proportion of more stable, saturated components. These results establish that while all three strains can utilize mixed waste streams, *A. oryzae* supplemented with 10% fresh oil provides the most effective biological platform for producing a nutritionally enhanced with a PUFA-enriched lipid profile.

### 3.2. Enzyme Activity

#### 3.2.1. Protease Activity

To evaluate the statistical significance of the observed variations, a post hoc Fisher’s Least Significant Difference (LSD) test was performed. Table 3 summarizes the statistical analysis of the specific enzyme activities across the different filamentous fungi strains and lipid supplementation sources (fresh and spent oil). The statistical matrix confirms significant differences (*p* < 0.05) in enzyme production, demonstrating that both the selection of the specific fungal strain and the type of oil supplementation significantly modulate the enzymatic profile during solid-state fermentation.

**Figure 1:**
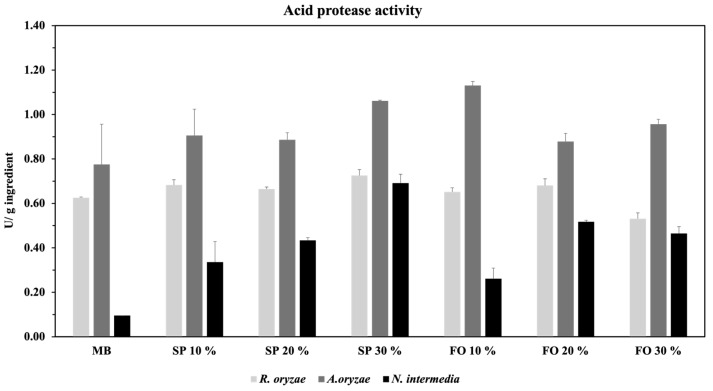
Endogenous acid protease activity (U/g of dry matter) of fungal biomass cultivated on bread waste under the experimental design (pH 4.0). MB: mycelium bread; FO: fresh oil; SP: spent oil. For a detailed intra-strain significance analysis across different oil concentrations, please refer to Table 3.

Acid protease activity varied significantly among fungal strains and oil supplementation levels, with all fermented treatments exhibited measurable enzyme activity (Figure 1). Among the three strains, *A. oryzae* consistently demonstrated the highest acid protease activity across all conditions, reaching its overall maximum of 1.13 U/g under 10% fresh oil supplementation. *R. oryzae* maintained stable acid protease activity levels between 0.53 and 0.72 U/g. In contrast, *N. intermedia* displayed fluctuations depending on the oil percentage and oil type, although all supplemented treatments exhibited significantly higher activity compared to the oil-free mycelium-bread (MB). With increasing spent oil supplementation, the acid protease activity of *N. intermedia* increased, reaching 0.69 U/g compared to 0.09 U/g in MB. Conversely, fresh oil additions exceeding 20% had no effect on the acid protease activity of *N. intermedia*.

The consistently high protease activity observed in *A. oryzae* reflects its well-established role as its proteolytic capacity for strong extracellular enzyme secretion during solid-state fermentation [70]. This performance is supported by the fact that *A. oryzae* possesses more secretory proteinase genes that function in acidic pH, reflecting its adaptation to such environments during domestication [71]. Furthermore, it has been reported that *A. oryzae* generally exhibits higher protease activity than *R. oryzae* in fungal biotechnology perspectives [72]. In contrast, the acid protease activity in *N. intermedia* is in opposition to its superior crude protein yield reported in MB (Section 3.1.1), highlighting a highly efficient nitrogen utilization for mycelium development instead of extracellular enzyme synthesis. This aligns with the observation that *N. intermedia* is more notable for its cellulolytic machinery than for its proteases, which enables efficient waste-to-food conversion [73]. However, the further increased level of activity at 30% spent oil indicates that oil quality critically influences enzyme regulation, pre-oxidized or partially degraded components in the spent oil may induce distinct metabolic stress responses or assimilation pathways compared to fresh triacylglycerols.

**Figure 2:**
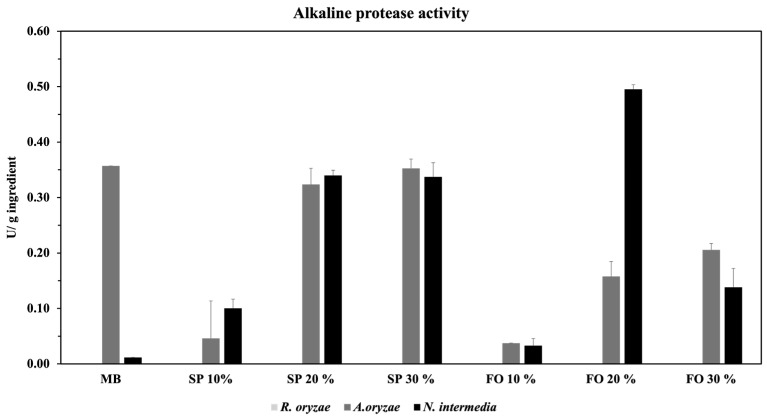
Endogenous alkaline protease activity (U/g of dry matter of ingredient) of fungal biomass cultivated on bread waste under the experimental design (pH 8.0). MB: mycelium bread; FO: fresh oil; SP: spent oil. For a detailed intra-strain significance analysis across different oil concentrations, please refer to Table 3.

Alkaline protease secretion was even more pronounced. *R. oryzae* showed no detectable activity (Figure 2). This lack of activity may be attributed to its specific protease profile, as *R. oryzae* is known to secrete aspartic proteinases with reported optimum pH levels typically between 5.0 and 5.2 [74,75,76]. In contrast, both *A. oryzae* and *N. intermedia* exhibited strong alkaline proteases activity specifically under spent oil supplementation, with spent oil exerting a statistically significant stimulatory effect (Table 3). For *A. oryzae*, this is consistent with its alkaline protease system, which has an optimum pH of 7.5 [77]. This pattern is best interpreted not as a response to protein residues in the oil, but rather to oxidation products generated during frying, including free fatty acids, aldehydes, peroxides, and short-chain volatiles [78]. These compounds are known to elicit mild oxidative or membrane stress in fungi, triggering the secretion of alkaline proteases as part of a general stress-response pathway [69].

#### 3.2.2. Chitinase Activity

**Figure 3:**
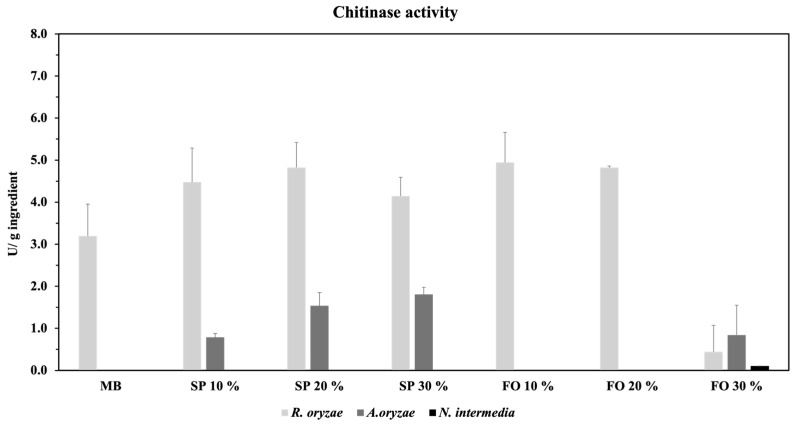
Endogenous chitinase activity (U/g of dry matter) of fungal biomass cultivated on bread waste under the experimental design (pH 5.0). MB: mycelium bread; FO: fresh oil; SP: spent oil. For a detailed intra-strain significance analysis across different oil concentrations, please refer to Table 3.

Chitinases are considered multifunctional enzymes that are critical for fungal growth and hyphal development [75]. As illustrated in Figure 3, *R. oryzae* demonstrated high chitinase activity in the oil-free control. In contrast, the other two species exhibited no detectable activity. Upon oil supplementation, there were no significant differences (Table 3) in the chitinase activity of *R. oryzae*; however, supplementation with 30% fresh oil caused a depression in chitinase activity. *N. intermedia* did not exhibit any chitinase activity across all treatments.

Potential roles of fungal chitinases include the degradation of exogenous chitin sources as well as the autolysis of cell walls from dead hyphal fragments for use as nutrient sources [79]. This autolysis facilitates cell wall remodeling during hyphal growth, branching, and defence against other fungi [80]. Specifically, the *R. oryzae* genome contains four protein models belonging to family GH20 and members of family CE9 (which includes N-acetylglucosamine-6-phosphate deacetylases) both of which are essential for the metabolism of chitin [81]. Consequently, the high chitinase activity in *R. oryzae* is supported by its ability to produce extracellular chitinases [82] and is induced by the autolysis of chitin within the hyphae of this fungus. In contrast, the Ascomycota species *N. intermedia* and *A. oryzae* demonstrated low to negligible chitinase activity. For *N. intermedia*, this is consistent with the fact that there is currently no information available regarding the determination of chitinase activity in fermentations with this strain.

However, *A. oryzae* exhibited increasing chitinase activity with higher spent oil supplementation, whereas fresh oil addition did not induce activity. In the CAZy database, a GH family 20 gene from *A. oryzae* (GenBank: BAE58709.1) has been inferred as a β-N-acetylhexosaminidase by homology, which indicates the potential to hydrolyze chitin [83]; however, these results suggest that this potential is only manifested under specific conditions. Since chitinase activity is induced by exogenous components and regulated by developmental stimuli, the spent oil used for supplementation in this study may contain impurities that triggered chitinase activity in *A. oryzae*. Nevertheless, further analyses are needed to detect the specific composition of the spent oil and determine the inducing effect of these contaminants on chitinase activity.

#### 3.2.3. Lipase Activity

**Figure 4:**
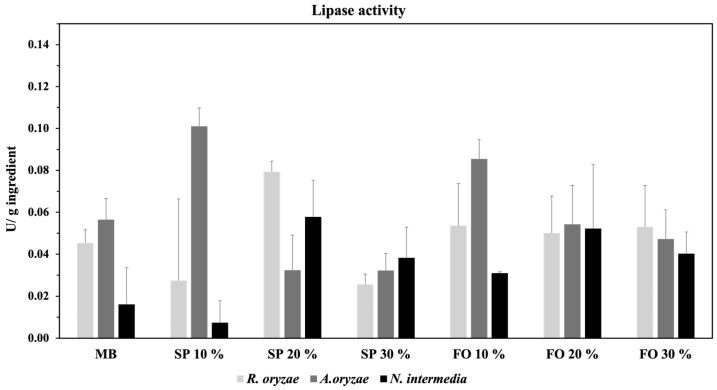
Endogenous lipase activity (U/g of dry matter of ingredient) of fungal biomass cultivated on bread waste under the experimental design (pH 8.0). MB: mycelium bread; FO: fresh oil; SP: spent oil. For a detailed intra-strain significance analysis across different oil concentrations, please refer to Table 3.

Lipases catalyze the hydrolysis of triglycerides into free fatty acids and glycerol, playing a central role in lipid accumulation, degradation, and fatty acid reconstruction in filamentous fungi [25]. The changes in lipase activity across the varying treatments, compared to MB for each strain, are illustrated in Figure 4. In the oil-free treatment, *R. oryzae* and *A. oryzae* exhibited significantly higher lipase activity compared to *N. intermedia*. Supplementation with 10% oil (both fresh and spent) induced lipase activity across all three fungal strains. Notably, *A. oryzae* achieved the highest overall lipase activity of 0.1 U/g under spent oil supplementation. Increasing fresh oil to 20% decreased the lipase activity of *A. oryzae* to 0.06 U/g, with no significant differences observed following further oil additions up to 30%. *R. oryzae* maintained consistent activity levels of approximately 0.05 U/g across all fresh oil treatments, whereas its highest significant lipase activity was achieved with 20% spent oil supplementation.

*A. oryzae* is well documented for its robust lipase activity, which has led to widespread industrial application in the food, pharmaceutical, and biofuel sectors [84]. Environmental conditions, such as temperature, pH, and nutrient availability, significantly impact lipase activity in *A. oryzae*. Optimal lipase production for this species has been reported at a pH of 5.5 and a temperature of 30 °C [85]. These optimal conditions likely contributed to the high lipase activity observed for *A. oryzae* in the oil-free treatment compared to the other two strains. Furthermore, the observed changes in fatty acid composition, evidenced by an increased PUFA/SFA ratio that reached a maximum of 5.9 with 10% oil supplementation, aligns with the versatility of these species in modifying lipid structures.

Substrate composition including type, supplementation level, and moisture content plays a pivotal role in determining lipase activity during SSF. The high content of unsaturated fatty acids in sunflower oil, particularly linoleic acid (C18:2), has been reported to effectively stimulate lipase production in some fungal species such as *Rhizopus chinensis* [86]. Across all evaluated species, lipase production declined by 30% oil supplementation. This inhibitory effect is consistent with previous reports indicating that excessive hydrophobic substrates create a physical barrier, which reduces oxygen transfer and suppresses energy-intensive enzyme synthesis [38].

## 4. Conclusions

This study demonstrates the feasibility of co-valorizing stale bread and spent sunflower oil through solid state fermentation using food-grade filamentous fungi, contributing to a circular nutrient recovery. The bioprocess yielded nutrient-rich mycelium-based products, with *N. intermedia* establishing its specialization as a superior protein producer (up to 36%). Enzymatic analysis provided a mechanistic foundation for these observations, highlighting *R. oryzae*’s structural modification driven by high chitinase activity and *A. oryzae*’s superior lipid restructuring facilitated by potent lipase induction at 10% sunflower oil supplementation level. Notably, the quality of the lipid source emerged as a critical determinant of biomass quality. While fresh oil supported the highest polyunsaturated fatty acid enrichment, spent oil served as a stronger inducer for alkaline proteases and lipases, despite the lower PUFA fractions. These findings provide a biological strategy for upcycling heterogeneous food waste into high-value functional ingredients, addressing the challenges with recourse recovery and provision of sustainable alternative nutrient sources for food and feed systems.

## Figures and Tables

**Table 1:** Composition of mycelium-bread according to the strains, oil type and level of supplementation including crude protein, starch, crude fat and crude fiber (g/100 g DM). Different lowercase and uppercase letters within the same column indicate statistically significant differences (*p* < 0.05) based on ANOVA followed by the corresponding post hoc test.

Species	Oil Type	Oil Supplement (%)	Crude Protein	Crude Fiber	Crude Fat	Starch
*A. oryzae*	-	0	22.2 ± 0.6 ^f^	31.7 ± 0.7 ^A^	3.2 ± 1.3 ^k^	6.5 ± 0.3 ^JK^
	Fresh	10	26.3 ± 0.1 ^cd^	32.0 ± 1.8 ^A^	6.4 ± 0.4 ^ijk^	12.3 ± 0.2 ^F^
		20	22.0 ± 0.7 ^f^	24.1 ± 1.7 ^A-C^	17.3 ± 0.7 ^fg^	7.7 ± 0.2 ^HIJK^
		30	18.9 ± 0.3 ^g^	23.2 ± 0.9 ^A-D^	25.9 ± 1.0 ^cde^	5.2 ± 0.4 ^K^
	Spent	10	24.8 ± 1.0 ^de^	31.1 ± 2.1 ^A^	5.9 ± 0.2 ^jk^	10.7 ± 0.0 ^FG^
		20	21.5 ± 0.9 ^f^	24.7 ± 8.0 ^A-C^	13.0 ± 0.8 ^gh^	8.1 ± 0.2 ^HIJK^
		30	19.0 ± 0.1 ^g^	27.3 ± 8.7 ^AB^	31.4 ± 0.7 ^bc^	7.7 ± 0.0 ^HIJK^
*R. oryzae*	-	0	26.6 ± 0.4 ^cd^	20.6 ± 0.6 ^A–F^	6.9 ± 0.8 ^ijk^	21.9 ± 0.3 ^DE^
	Fresh	10	18.2 ± 0.6 ^g^	10.3 ± 2.7 ^E–G^	17.6 ± 0.0 ^fg^	27.5 ± 0.1 ^AB^
		20	16.7 ± 0.5 ^h^	10.1 ± 1.1 ^E–G^	25.6 ± 0.3 ^de^	28.8 ± 0.3 ^A^
		30	12.5 ± 1.0 ^j^	7.7 ± 2.4 ^G^	34.9 ± 0.4 ^ab^	25.8 ± 0.4 ^BC^
	Spent	10	17.6 ± 0.0 ^gh^	22.1 ± 3.5 ^A–E^	19.7 ± 0.2 ^f^	25.1 ± 0.0 ^BC^
		20	15.4 ± 0.7 ^hi^	13.5 ± 3.7 ^C–G^	28.4 ± 1.2 ^cd^	24.3 ± 0.4 ^CD^
		30	14.2 ± 0.9 ^ij^	14.2 ± 0.9 ^FG^	37.3 ± 1.2 ^a^	21.3 ± 0.2 ^E^
*N. intermedia*	-	0	36.0 ± 0.3 ^a^	24.1 ± 0.7 ^A–C^	3.3 ± 0.3 ^k^	6.5 ± 0.1 ^JK^
	Fresh	10	31.1 ± 0.4 ^b^	20.6 ± 1.0 ^A–F^	11.6 ± 0.2 ^hi^	9.0 ± 0.1 ^GHI^
		20	22.1 ± 0.3 ^f^	15.8 ± 2.8 ^B–G^	17.3 ± 0.7 ^f^	8.4 ± 0.0 ^JK^
		30	18.0 ± 0.5 ^g^	11.6 ± 3.0 ^D–G^	39.0 ± 0.1 ^a^	7.5 ± 0.4 ^HIJK^
	Spent	10	27.6 ± 0.8 ^c^	16.6 ± 0.5 ^B–G^	11.2 ± 0.0 ^hij^	9.4 ± 0.0 ^GH^
		20	23.6 ± 0.5 ^ef^	18.0 ± 0.5 ^B–G^	23.7 ± 1.5 ^ef^	8.8 ± 0.0 ^GHIJ^
		30	16.8 ± 0.6 ^h^	11.0 ± 0.8 ^D–G^	36.3 ± 1.1 ^ab^	6.7 ± 0.2 ^IJK^
Uninoculated control	-	0	11.50 ± 0.50	1.50 ± 0.50	2.00 ± 0.10	64.90 ± 2.02

**Table 2:** Fatty acid type and distribution (% of total fatty acids) of the fermented biomass. SFA: Saturated fatty acids; MUFA: Mono-unsaturated fatty acids, PUFA: Poly unsaturated fatty acids. Different lowercase and uppercase letters within the same column indicate statistically significant differences (*p* < 0.05) based on ANOVA followed by the corresponding post hoc test.

Strain	Oil Type	Oil Supplementation (%)	SFA	MUFA	PUFA	PUFA/SFA
*A. oryzae*	-	0	12.2 ± 0.6 ^c^	34.4 ± 0.3 ^J^	53.3 ± 0.3 ^A^	4.3 ± 0.2 ^bc^
	Fresh	10	7.5 ± 0.6 ^ef^	48.5 ± 0.3 ^I^	44.0 ± 0.4 ^B^	5.9 ± 0.6 ^a^
		20	7.4 ± 0.0 ^ef^	54.5 ± 0.1 ^GH^	38.0 ± 0.0 ^CD^	5.1 ± 0.0 ^ab^
		30	7.8 ± 0.0 ^c–f^	57.7 ± 0.0 ^D–F^	34.5 ± 0.0 ^EF^	4.4 ± 0.0 ^abc^
	Spent	10	9.0 ± 0.4 ^c–e^	50.3 ± 1.8 ^I^	40.6 ± 2.2 ^C^	4.5 ± 0.5 ^abc^
		20	9.5 ± 0.0 ^cd^	53.7 ± 1.4 ^H^	36.8 ± 1.3 ^DE^	3.9 ± 0.1 ^abc^
		30	8.6 ± 0.2 ^c–f^	54.6 ± 0.1 ^GH^	36.8 ± 0.1 ^DE^	4.3 ± 0.1 ^abc^
*N. intermedia*	-	0	21.3 ± 0.0 ^a^	48.7 ± 0.2 ^I^	29.4 ± 0.0 ^I–K^	1.4 ± 0.0 ^c^
	Fresh	10	12.1 ± 0.0 ^b^	57.8 ± 0.1 ^C–F^	29.8 ± 0.1 ^H–K^	2.5 ± 0.0 ^abc^
		20	9.2 ± 0.5 ^c–e^	60.6 ± 0.4 ^A–C^	30.0 ± 0.1 ^H–K^	3.3 ± 0.2 ^abc^
		30	8.3 ± 0.2 ^c–f^	61.3 ± 0.2 ^AB^	30.2 ± 0.0 ^H–K^	3.6 ± 0.1 ^abc^
	Spent	10	12.6 ± 0.7 ^b^	57.3 ± 0.6 ^E–G^	29.9 ± 0.0 ^H–K^	2.4 ± 0.1 ^bc^
		20	11.5 ± 0.3 ^b^	59.4 ± 0.3 ^B–E^	28.8 ± 0.0 ^K^	2.5 ± 0.1 ^abc^
		30	11.7 ± 0.4 ^b^	59.6 ± 0.3 ^A–E^	28.5 ± 0.1 ^K^	2.4 ± 0.1 ^abc^
*R. oryzae*	-	0	13.3 ± 0.5 ^b^	31.4 ± 0.4 ^K^	55.3 ± 0.0 ^A^	4.2 ± 0.1 ^abc^
	Fresh	10	8.2 ± 0.5 ^c–f^	55.7 ± 0.8 ^F–H^	36.1 ± 0.2 ^DE^	4.4 ± 0.2 ^abc^
		20	7.4 ± 0.4 ^d–f^	60.2 ± 0.0 ^A–D^	32.2 ± 0.4 ^F–H^	4.2 ± 0.1 ^abc^
		30	7.1 ± 0.3 ^f^	60.7 ± 0.8 ^AB^	32.1 ± 0.5 ^G–J^	4.5 ± 0.0 ^abc^
	Spent	10	9.6 ± 0.3 ^c^	57.3 ± 0.2 ^D–G^	33.1 ± 0.0 ^FG^	3.5 ± 0.1 ^abc^
		20	9.6 ± 0.7 ^c^	58.5 ± 2.3 ^B–F^	31.9 ± 1.5 ^G–I^	3.3 ± 0.1 ^abc^
		30	8.4 ± 0.0 ^c–f^	62.4 ± 0.0 ^A^	29.3 ± 0.0 ^JK^	3.5 ± 0.0 ^abc^
Uninoculated control	-	0	12.03 ± 0.74	42.70 ± 1.96	45.44 ± 0.92	3.79 ± 0.16

**Table 3:** Statistical analysis of specific enzyme activities across different filamentous fungi strains and lipid supplementation sources. Values within the same column for “Oil type” (uppercase letters) or “Species” (lowercase letters) sharing at least one common letter do not differ significantly (*p* < 0.05).

	Acid Protease	Alkaline Protease	Chitinase	Lipase
Oil type				
MB	A	AB	AB	A
Fresh 10	B	B	A	A
Fresh 20	B	A	A	A
Fresh 30	B	AB	B	A
Spent 10	B	B	A	A
Spent 20	B	A	A	A
Spent 30	C	A	A	A
*p*-value	0.002	0.006	0.067	0.529
Species				
*R. oryzae*	a	b	a	ab
*A. oryzae*	b	a	b	a
*N. intermedia*	c	a	b	b
*p*-value	0.000	0.000	0.000	0.000

## Data Availability

The original contributions presented in this study are included in the article. Further inquiries can be directed to the corresponding author.
